# Epistasis Creates Invariant Sites and Modulates the Rate of Molecular Evolution

**DOI:** 10.1093/molbev/msac106

**Published:** 2022-05-16

**Authors:** Ravi Patel, Vincenzo Carnevale, Sudhir Kumar

**Affiliations:** 1 Institute for Genomics and Evolutionary Medicine, Temple University, Philadelphia, PA 19122, USA; 2 Department of Biology, Temple University, Philadelphia, PA 19122, USA; 3 Center for Excellence in Genome Medicine and Research, King Abdulaziz University, Jeddah, Saudi Arabia

**Keywords:** epistasis, neutral theory, potts model, purifying selection

## Abstract

Invariant sites are a common feature of amino acid sequence evolution. The presence of invariant sites is frequently attributed to the need to preserve function through site-specific conservation of amino acid residues. Amino acid substitution models without a provision for invariant sites often fit the data significantly worse than those that allow for an excess of invariant sites beyond those predicted by models that only incorporate rate variation among sites (e.g., a Gamma distribution). An alternative is epistasis between sites to preserve residue interactions that can create invariant sites. Through computer-simulated sequence evolution, we evaluated the relative effects of site-specific preferences and site-site couplings in the generation of invariant sites and the modulation of the rate of molecular evolution. In an analysis of ten major families of protein domains with diverse sequence and functional properties, we find that the negative selection imposed by epistasis creates many more invariant sites than site-specific residue preferences alone. Further, epistasis plays an increasingly larger role in creating invariant sites over longer evolutionary periods. Epistasis also dictates rates of domain evolution over time by exerting significant additional purifying selection to preserve site couplings. These patterns illuminate the mechanistic role of epistasis in the processes underlying observed site invariance and evolutionary rates.

## Introduction

Half a century ago, Uzzell and Corbin (1971) showed that the distribution of the number of substitutions over amino acid sites had a much larger dispersion than expected from the same evolutionary rate across all sites. That is, the frequency of sites with different numbers of substitutions did not follow the Poisson distribution expected if the evolutionary rate was the same across sites (single [S] rate model). A negative binomial distribution better describes the observed substitution frequencies spectrum across sites (SFSS), which can arise when the evolutionary rates vary from site to site and are drawn from a Gamma (G) distribution (Uzzell and Corbin 1971). Subsequently, it was reported that the number of completely conserved sites (invariant sites, I-sites) across sequences could significantly exceed those predicted by a Gamma distribution of rates (Gu et al. 1995). A mixture model (I + G) containing a class of I-sites alongside a Gamma (G) distribution of site rates often fits the observed SFSS much better, e.g., Yang (1993) and Kumar (1996). The I + G rate model is frequently used in molecular evolutionary analyses.

The phenomenon of excess of I-sites can arise due to site-specific amino acid preferences to conserve function (Fitch and Margoliash 1967; Kimura and Ohta 1974; Doud et al. 2015). Two contrasting possibilities are that 1) sites evolve largely independently with site-specific amino acid preferences exerting negative selective pressure (independent evolution model, IE model), and 2) substitutions at a site depend on amino acid residues found at other sites due to intramolecular couplings (coupled evolution model, CE model). The IE and CE models are not mutually exclusive because site-specific preferences for amino acid residues can be a byproduct of CE or occur along with site-wise couplings. The fundamental difference between the two alternatives is that the purifying selection operates directly and independently on individual residues in the IE model, whereas purifying selection operates to preserve epistasis among coupled sites in the CE model.

**Fig. 1.**
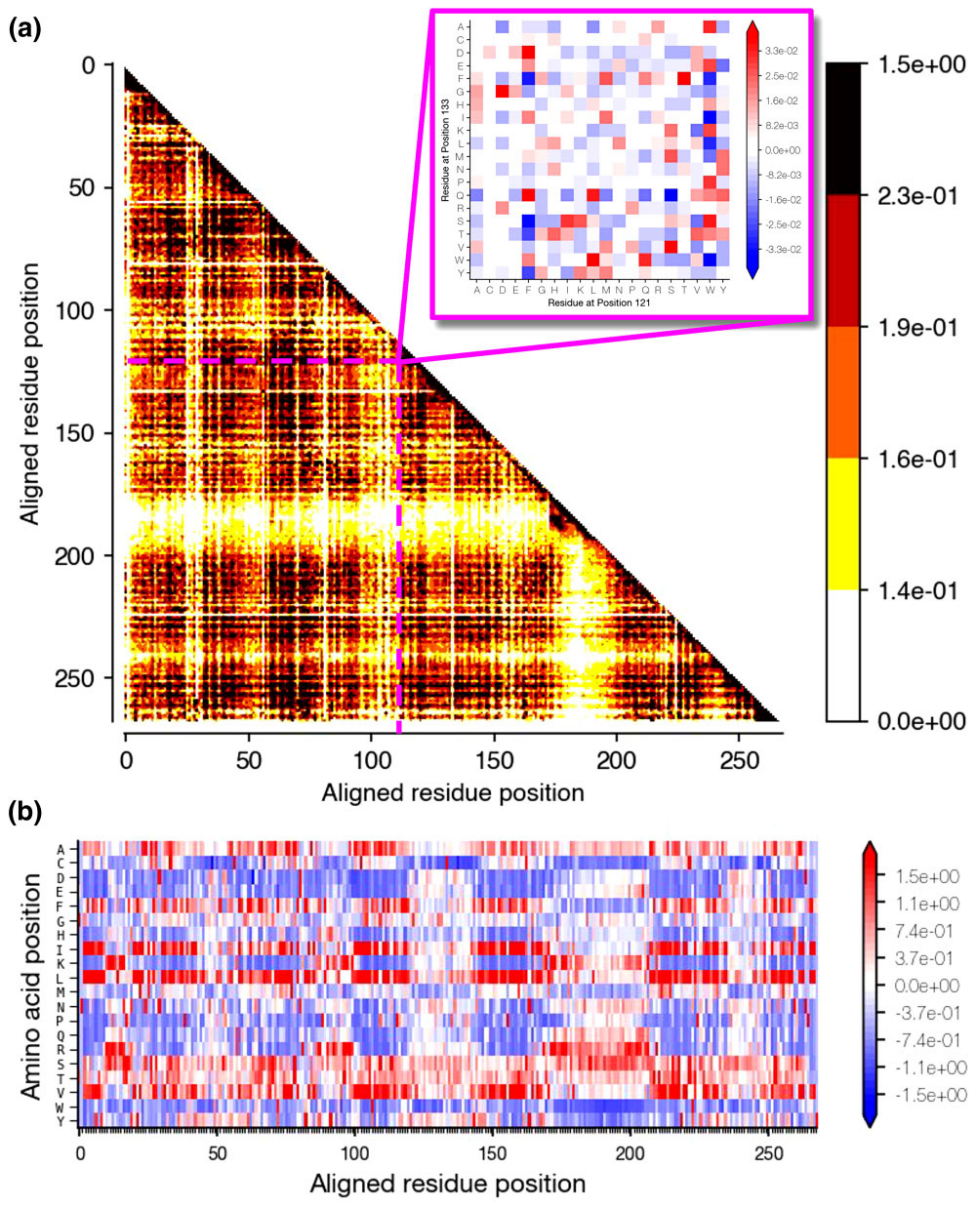
Pairwise coupling and local preference matrices for PF00001. (*a*) Residue–residue preferences for each pair of aligned positions within a sequence. (*b*) Local site-specific amino-acid preferences. In panel a, two dimensions visualized here represent a pair of sites in a sequence. The other two dimensions (inset) specify the coupling strength for specific residues at each site in the pair. Each cell of the larger matrices shows the Frobenius norm of the couplings among all residues at a given pair of sites. Darker, warmer colors represent stronger overall coupling between sites. The inset shows the coupling between 20 residues for the given pair of sites; more positive values indicate a more preferred combination and more negative values indicate a more undesired combination of residues. Zero values indicate that a specific combination of residues is neither favored nor rejected.

Potts models have been used for modeling CE (Shekhar et al. 2013; Couce et al. 2017; Gao et al. 2019; de la Paz et al. 2020). These models incorporate the strength of coupling between sites as well as more traditional individual site residue preferences. Direct coupling analysis (DCA) of large sequence alignments for protein domain families has been employed to estimate pairwise coupling constraints among all positions (Weigt et al. 2009). fig. 1 shows the DCA-inferred parameters of a Potts model for a protein domain derived from an alignment of thousands of sequences across the tree of life and genomes. The relative strength of *pairwise* epistatic coupling between any two positions is reflected in the color intensities of cells in fig. 1*a*. Each cell in the pairwise couplings matrix further consists of a 21 × 21 matrix (20 amino acid characters and one indel “−” character) whose coefficients reflect the probability of observing each given pair of amino acids among all sites (fig. 1*a* inset).

The Potts model also includes additional terms corresponding to equilibrium frequencies of amino acids at each position (fig. 1*b*). Importantly, site-specific residue preferences at a given time in the evolution of a domain are contextual in the Potts model, as they depend on specific residues present in other positions (Weigt et al. 2009; see *Methods*). Due to the formal analogy between Potts model probability and Gibbs-Boltzmann distribution, the log-likelihood is referred to as statistical or Hamiltonian energy and can be interpreted as an indirect measure of evolutionary fitness. *In vivo* assays of *in silico* evolved sequences show correlations between the biological fitness and Hamiltonian Energy for various enzymes in *Escherichia coli* (Russ et al. 2020; Bisardi et al. 2021). These observations support the use of the Potts model to study the impact of intramolecular epistasis on molecular sequence evolutionary patterns in protein domains (Rizzato et al. 2019; de la Paz et al. 2020; Patel and Kumar 2021).

In simulation studies using epistasis during sequence evolution, Rizzato et al. (2019) showed that epistasis creates heterogeneity of observed substitution rates among sites. Soon after, de la Paz et al. (2020) developed an extended simulation framework and showed that the overdispersion of evolutionary rates among evolutionary lineages and sites within domains are emergent properties of epistasis. Using de la Paz et al. (2020) framework, Patel and Kumar (2021) found that epistasis creates I-sites more readily than the Gamma-distributed rate variation among sites at biologically realistic evolutionary divergences.

While Patel and Kumar (2021) showed epistasis (CE model) to be one natural source of I-sites, the contribution of site-specific amino acid preferences alone without site couplings (IE model) is yet to be evaluated and contrasted with the CE model. This is important because site-specific preferences induce strong purifying selection at a position independent of pairwise residue constraints (Doud et al. 2015). Therefore, we used de la Paz’s sequence evolution with epistatic constraints (SEECs) simulation framework to simulate coupled and non-coupled substitutions to dissect the net effect of site interactions on the creation of I-sites and exertion of purifying selection in protein evolution. We report that site couplings are more influential in maintaining I-sites than site-specific amino acid residue preferences. However, together, they provide natural mechanistic explanations for many evolutionary I-sites in protein domains and dictate their evolutionary rate.

## Results

### Simulating Protein Sequence Evolution

We employed de la Paz et al.’s framework (2020) that uses a Potts model to simulate SEECs. It simulates the evolution of protein sequences in a stepwise manner in which each step is a generation that involves choosing a position at random and then attempting an amino acid substitution. The amino acid to be substituted is randomly selected from a conditional probability distribution calculated using each residue’s relative contribution to the overall sequence fitness (statistical Hamiltonian Energy). In this substitution process, pre-existing residues at all other positions provide the context for the new substitution and dictate the probability of substitution at the selected position based on the parameters in the Potts model. A position selected for substitution may not receive a change because the residue selected for substitution is not allowed by the Potts model. This site would be considered invariant for as long as its amino acid found in the first generation is not changed (see *Methods*).

We used SEEC to simulate protein sequence evolution under a CE model that includes pairwise epistatic constraints *and* individual site-specific residue preferences, IE model with no pairwise epistasis but *only* individual site-specific preferences, and a uniform evolution (UE) model of *neither* pairwise epistasis *nor* site-specific preferences. The UE model served as a null model of molecular evolution where all amino acid residues had an equal probability of substitution at all positions independent of the sequence at all other positions. There were no other sources of negative or positive selection (strictly neutral evolution). Under the UE model, the number of substitutions observed in a sequence is purely a function of the amount of time elapsed and the stochasticity of the evolutionary process. Thus, the numbers of substitutions and I-sites observed in a sequence under the UE model are simply due to mutational input that create amino acid substitutions, which is the baseline to generate the net contribution of CE and IE models.

Adjusting CE and IE models for expectations provided by the null UE simulations results in a simplified framework where we can tease apart the contributions of site-specific and pairwise coupling effects on the patterns of substitutions observed during protein domain evolution. Differences in substitution patterns in CE simulations versus UE expectations at each site are attributed to the combination of pairwise site interactions (ε) and local site-specific residue preferences (*ℓ*), which we term combined effects (ɛ = ε + *ℓ* ⇔ CE − UE simulations). Similarly, differences between IE and UE simulations show substitutions due solely to local site-specific residue preferences (local effects; *ℓ* ⇔ IE − UE simulations). Thus, the difference between combined (ɛ) and local (*ℓ*) effects provides the net substitutions and I-sites contributed by pairwise epistasis only (ε = ɛ − *ℓ*), i.e., no local site-specific residue preferences.

In all simulations, we tracked unsubstituted sites to count the number of I-sites during SEEC runs. We also counted the number of total substitutions in the sequence to estimate the evolutionary rate and, thus, the degree of negative selection pressure due to *ℓ* and ɛ. We analyzed ten protein domain models inferred via DCA by de la Paz et al. (2020). They spanned diverse sequences, structures, and biochemical compositions.

### Excess of I-sites Created by Site Interactions

**Fig. 2.**
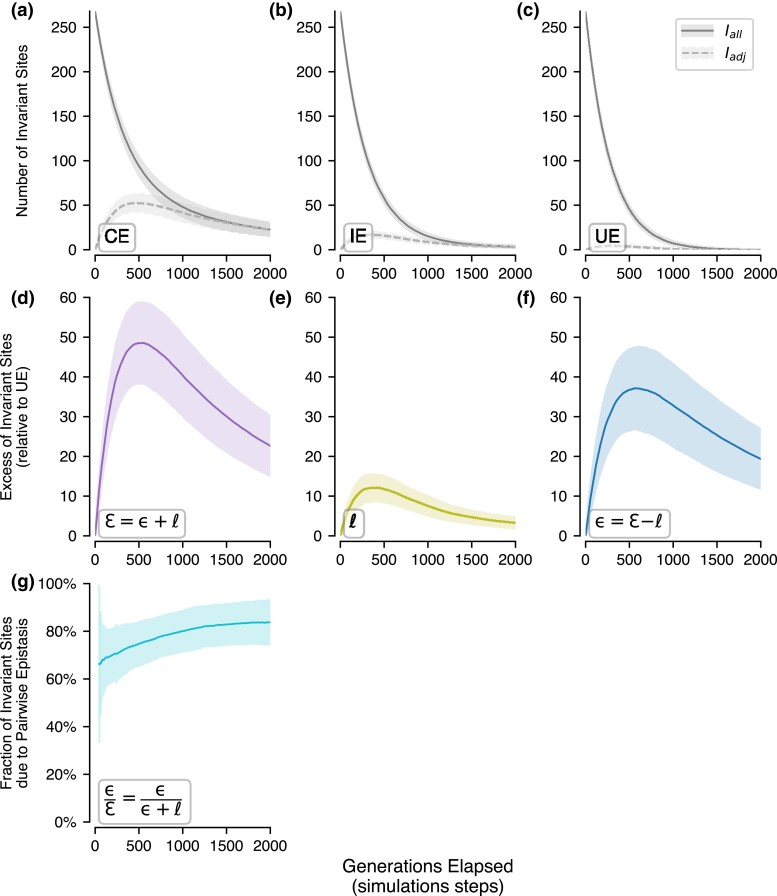
The number of I-sites observed in PF00001 by generation. Distributions of sites with no substitutions are shown for the first 2,000 generations of sequence evolution (simulation steps; *x*-axis), aggregated across 500 simulation replicates. Measurements are shown for when all I-sites (*I*_all_; solid line) are counted and when only sites that have been tested at least one time (*I*_adj_; dashed line) are counted (see “Simulations” in Methods). The mean (solid lines) and one standard deviation (colored spans) of measurements across replicates are shown for each generation. The number of I-sites at each generation is shown for (*a*) CE: pairwise epistasis and local fields, (*b*) IE: local fields only, and (*c*) UE: neither epistasis nor local fields. The excess of I-sites created during sequence evolution due to (*d*) combined effects, ɛ, and (*e*) local effects, *ℓ*, are calculated by subtracting null UE expectations from CE and IE simulations. (*f*) shows the number of I-sites due to pairwise epistasis (ɛ). Additionally, we calculate (*g*) the proportions of I-sites created due to ɛ only over time.

We first examined the number of I-sites observed during domain evolution. We compared the number of sites that remained invariant at increasingly later generations for each set of simulation parameters, corresponding to more time elapsed. As more substitutions were attempted in a domain over time, the number of I-sites decreased for CE and IE simulations (fig. 2*a* and *b*, solid line). However, the decay rate of I-sites under the CE model (fig. 2*a*) was much slower than that observed in IE simulations (fig. 2*b*). These rates were lower than those for UE simulations (fig. 2*c*).

In addition to all the I-sites, we tracked the number of sites that remained invariant despite being selected for amino acid substitution(s). That is, the attempted substitutions experienced negative selection. Dashed lines in fig. 2 show these trends (panels *a*–*c*), which are also recaptured in panels *d*, *e*, and *f*. The combined effect of pairwise couplings and local site-specific residue preferences, ɛ, for PF00001 created on average ∼16% *more* I-sites between 250 and 1000 generations of CE sequence evolution (fig. 2*d*, solid curve). In contrast, local effects, *ℓ*, for PF00001 created an average excess of 3.7% I-sites for the same periods of IE simulations (fig. 2*e*, solid curve).

**Fig. 3.**
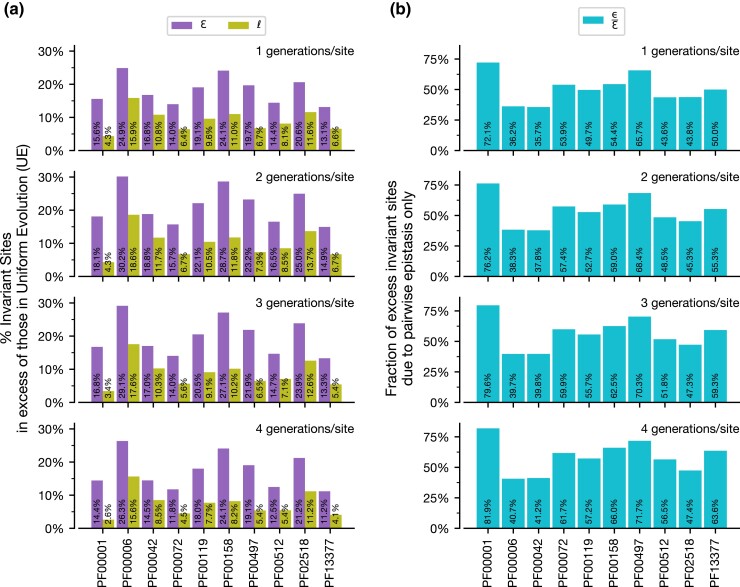
Excess I-sites relative to UE over time. (*a*) The fraction of sites that remain unsubstituted due to combined effects of pairwise epistasis and local site-specific residue preferences (ɛ, purple) and local site-specific preferences only (*ℓ*, green). (*b*) Excess I-sites due to pairwise epistasis only relative to the combined effects (ε/ɛ, cyan) are shown at four increasing longer evolutionary histories (1.0–4.0 generations/site) for all ten protein domains analyzed.

Fewer excess I-sites were created due to ɛ and *ℓ* at earlier and later generations than intermediate generations (figs. 2*d* and 2*e*). This is expected because earlier in an evolutionary trajectory, too few substitutions have occurred to differentiate between UE and CE (or IE) models. Later in an evolutionary course, too many substitutions have occurred for many sites to remain invariant. We find similar trends in the nine other protein domains analyzed (fig. 3*a*). Therefore, evolutionary divergence spanned in a comparison is a major determinant of the proportion of I-sites.

We then determined the excess of I-sites due solely to pairwise site-interactions, ε (fig. 2*f*). While the excess of I-sites created solely due to site-interactions is at a maximum at intermediate generations, we find that the mean proportion of I-sites due to ε (ε/ɛ) increases consistently from ∼70.7% to ∼80.0% over sequence evolution (fig. 2*g*). This indicates that local site-specific residue preferences play a small but consistent role in maintaining I-sites over longer evolutionary periods. For the nine other protein domain families analyzed, ɛ values varied, but the general trend also holds (fig. 3*b*).

Across analyses of 10 protein domain families, we see local site-specific residue preferences (*ℓ*) can produce a sizable amount of I-sites (fig. 3*a*) and, in many cases, create the majority of such sites, but, overall, site-couplings (ε) always add substantially to this collection.

### Site Couplings Modulate the Rate of Domain Evolution

Using domain sequences produced using SEEC, we compared the degree of negative selection imposed by 1) pairwise epistatic couplings, ε and 2) local site-specific residue preferences, *ℓ*. Positions with less constraint will accept more substitutions due to less purifying selection, while those with greater constraints will accept fewer substitutions due to more purifying selection.

**Fig. 4.**
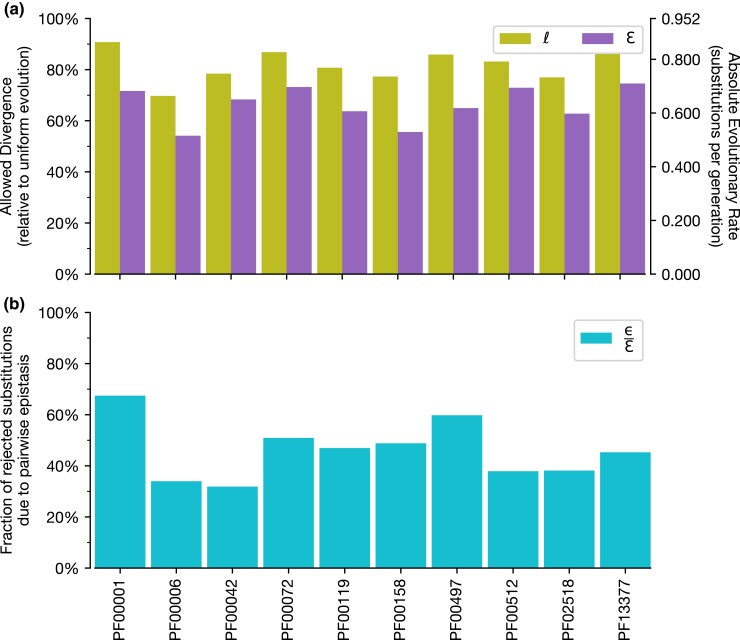
Allowed evolutionary divergence for constrained sequence evolutions. (*a*) Rates of evolution for IE (due to *ℓ*; green) and CE (due to ɛ; purple) are shown for all ten protein domain families analyzed. “Allowed divergence” (left axis) is a ratio of evolutionary rate for a given model relative to the null, UE evolutionary rate. Absolute rates of evolution are included on the right axis. (*b*) The fraction of negative selection due to pairwise epistasis relative to that from combined effects (ε/ɛ; blue).

We analyzed the rate of evolution in simulations for the ten protein domain families. The evolutionary rate was measured as the number of substitutions per generation and adjusted to ensure that the same amount of mutational input was experienced by all ten protein domain families of different lengths; substitutions per generation = (substitutions/site)/(generations/site). Protein sequences undergoing UE do not experience negative selection, as all constraints from epistatic coupling and local residue preference are absent. Thus, sequences for all protein domain families show the same evolutionary rate: 0.952 substitutions per generation. Despite a uniform probability distribution used to select amino acids for substitution in each generation of the simulation, the UE evolutionary rate is not 1.0 substitutions per generation. This is because there is only a 95.2% chance of an amino acid being substituted when a replacement is chosen randomly with equal probability. Thus, on average, every one in 21 generations, the same amino acid as that of the previous generation, will be selected (see “Simulating protein sequence evolution”) simply by chance, resulting in an “unsubstituted” position. Thus, in the absence of purifying selection (UE), simulated protein sequences are expected to allow 20/21 substitutions per generation (21 choices; 20 amino acids + 1 “−” alignment gap character). Negative selection pressures imposed by sequence constraints from pairwise epistasis (ε) and local residue preferences (*ℓ*) will proportionally decrease the evolutionary rate below the null UE rate. The rates of evolution for the ten protein domain families analyzed (fig. 4*a*, right axis) using IE were as low as 0.66 and high as 0.86 substitutions per generation, while the evolutionary rates using CE are found to be between 0.52 and 0.71 substitutions per generation.

**Fig. 5.**
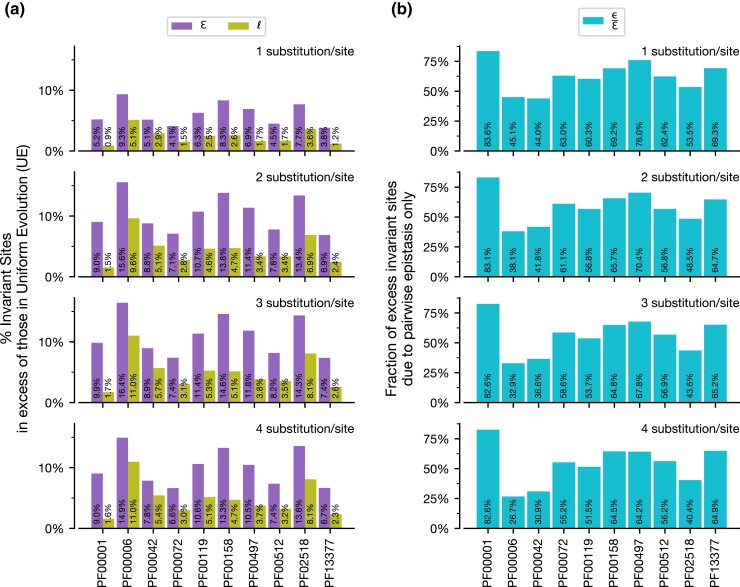
Excess I-sites over UE over time at different evolutionary distances. (*a*) The fraction of sites that remain unsubstituted due to combined effects of pairwise epistasis and local site-specific residue preferences (ɛ, purple) and local site-specific preferences only (*ℓ*, green). (*b*) Excess I-sites due to pairwise epistasis only relative to the combined effects (ε/ɛ, cyan) are shown at four increasing sequence divergences (1.0–4.0 substitutions/site) for all ten protein domain families analyzed.

Based on these expected rates of evolution for the protein domain sequences, we determined the extent of purifying selection during sequence evolution due to pairwise epistasis and local site-specific residue preferences. We measured this effect as an evolutionary rate ratio with the constrained model (CE or IE) to that for the unconstrained null, UE. We refer to this ratio as the *allowed divergence* for a given model, showing how much sequence change was allowed relative to that expected in unconstrained evolution. The complement of allowed divergence thus provides an estimate of the amount of sequence change *prevented* due to negative selection purging unacceptable amino acid replacement mutations. For example, evolution of PF00001 under IE was found to have 90.8% allowed divergence (fig. 5*a*, left axis) (i.e., IE/UE = 0.864/0.952 = 90.8%). Here, purifying selection due to local site preferences (*ℓ*) prevented 9.2% (100% allowed under UE − 90.8% allowed under IE) of potential amino acid replacements that would otherwise be found as substitutions (purged replacement mutations). PF00001 evolution under CE allowed even less divergence, 71.7%, with purifying selection removing 28.3% of tested replacement mutations. So, the combined effect (ɛ = ε + *ℓ*) of pairwise epistasis and local residue preferences in the PF00001 domain evolution resulted in >19% (19.1% = 28.3% − 9.2%) purifying selection relative to that caused by local residue preferences (*ℓ*) under IE alone. Examination of the other protein domain families showed similar results (fig. 4*a*).

We also calculated the amount of purifying selection due solely to pairwise epistasis (ε/ɛ) as the fraction of replacement mutations rejected under the combined effects (ɛ) of the CE model that were not due to local effects (ɛ − *ℓ* = ɛ). The component of purifying selection imposed solely due to pairwise epistasis ranged from 31.9% to 67.5% (fig. 4*b*) among the ten protein domains analyzed. So, while IE leads to negative selection during sequence evolution due to site-specific residue preferences, the addition of pairwise epistasis in CE produces a considerable amount of negative selection as well, in many cases generating the majority of negative selection pressures during protein sequence evolution.

### Pairwise Epistasis Directs Substitution Patterns Creating I-sites

The evolutionary rate of a protein sequence reflects the amount of negative selection constraining its evolution. Positions that are more constrained are expected to accumulate fewer substitutions (larger fraction of purged mutations) over time than positions that are less constrained. Thus, the number of unsubstituted sites in a protein sequence will vary with evolutionary time elapsed (see “Site couplings modulate the rate of domain evolution” and fig. 4) and with the degree of constraints across the entire sequence.

So, to adjust for the impact of differential negative selection across positions on the number of I-sites due to variation in constraints between CE and IE evolution, we examined the amount of excess I-sites created by epistasis under CE compared to IE having experienced the same amount of sequence divergence (substitutions per site). If pairwise epistasis did not impose additional sequence constraints that differed from constraints due to local site-specific residue preferences only, then we would expect both CE and IE to have the same number of I-sites after having accumulated the same number of substitutions, regardless of how much time is required to do so in each case.

**Fig. 6.**
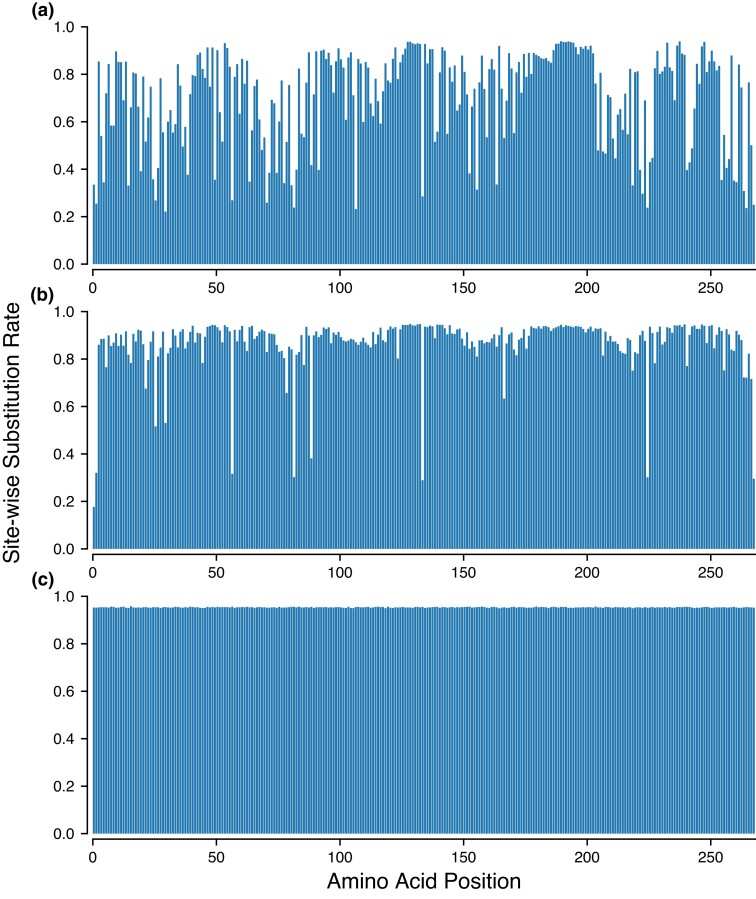
Mean substitution rates across sites for PF00001 derived from 500 simulation replicates. The substitution rate (*y*-axis) for each of 268 amino acid positions (*x*-axis) is calculated as the average number of generations for which a site remains invariant (substitution rate = time between possible substitutions/time between accepted substitutions). It provides an insight into how long a site is expected to remain invariant in independent evolutionary runs under an (*a*) CE model, (*b*) IE model, and (*c*) UE model.

Here, we compared the excess of I-sites observed in CE evolution and IE evolution for each protein domain family, but at a fixed sequence divergence (substitutions per site) instead of evolutionary time (generations per site). We found that sequence evolution under CE still created more I-sites than IE (fig. 5*a*). In fact, for seven of the ten protein domain families analyzed, more than 50% of the I-sites created were due to pairwise epistasis (fig. 5*b*). Epistasis in the other three domains still contributed no less than 26.7% of I-sites. These patterns suggest that pairwise epistasis provides unique constraints that substantially change the patterns of substitution that cause decreased substitution rate per site under increased constraints (fig. 6), creating I-sites due to differential negative selection across sites in a protein domain.

## Discussion

Two evolutionary factors contributing to the occurrence of I-sites are lack of mutational input and purging of alleles by differential negative selection. If enough evolutionary time has not elapsed, biological and evolutionary forces will not have had enough opportunity to create and test the effective fitness of possible mutations against natural selection. Both highly and un-constrained positions would appear to be lacking substitutions, but the source of such unsubstituted, I-sites would be ambiguous. With sufficient and constant mutational input at longer evolutionary timespans, the I-sites are expected to be unsubstituted due to stronger negative selection at positions with stronger sequence constraints than at positions with weaker (or no) constraints.

Some targets of natural selection based on structural and functional constraints have previously been proposed and developed into models. For example, protein structures are expected to be constrained based on environmental factors like the cellular environment; the protein solvent then chemically restricts the regions of the protein exposed to its surface. Similarly, the “internal” protein environment dictates the residues needed to ensure contacts that stabilize a protein's tertiary structure. Structural constraints also arise from protein flexibility and folding requirements (see review Echave et al. 2016). Sites in protein sequences can be conserved due to biochemical and functional importance, such as those required for proper protein-substrate interaction and binding. Further, even at the organismal level, sequence constraints can induce natural selection, as seen when tRNA availability limits codon usage.

Non-structural constraints have also affected natural selection during protein sequence evolution. For example, the intensity and tissue-specificity of gene expression have been shown to directly correlate with reduced evolutionary rates of proteins, e.g., (Subramanian and Kumar 2004). Genomic composition of genes (e.g., length of intronic sequence) and length of a protein’s coding sequence (CDS) have also been associated with natural selection in sequence evolution, with more compact genes and shorter CDS having higher evolutionary rates, e.g., (Lipman et al. 2002; Liao et al. 2006). Thus, a wide variety of higher order biological features are expected to constrain protein sequence evolution. As the effects of higher-order constraints cascade through to the lower level sequence constraints, their effects cannot be statistically differentiated in pairwise-epistasis and site-specific amino acid preferences with protein sequence MSAs only. In fact, we can expect that even at the single-position level, site-specific preferences estimated using MSAs are not independent but partly a result of pairwise-epistatic constraints. Indeed, site-specific preferences can be attributed to maintaining sequence and structural properties for proper biological function. However, very few such properties are independent of upstream requirements: single sites dictate secondary and tertiary protein structure, which define protein–protein interactions, etc.

As we previously mentioned, however, DCA-based models have provided a useful snapshot of a mechanism underlying protein sequence evolution that can recreate various statistical properties of sequences observed in empirical datasets, including rate heterogeneity and the occurrence of I-sites. Using Potts statistical models that incorporate epistasis (pairwise positional sequence constraints), we can describe protein sequence evolution without additional explicit parameterization of changes in the natural selection over both sequence position and evolutionary time. In doing so, we find that pairwise epistatic constraints create variation in evolutionary rates both across positions and over time, more so than local site-specific constraints only. In fact, the importance of pairwise epistasis in affecting evolution increases relative to local amino acid preferences only with more sequence evolution (divergence). Highly constrained sites due to epistasis will remain unsubstituted over a large course of evolutionary time and classified as invariant. The number and positions of the I-sites are not constant, nor is the substitution rate of a given position constant in the presence of epistasis. Because Potts models provide an overarching statistical model for sequences of a protein domain family with a shared biological function, we can see that this change in evolution rates (and thus, site invariance state) over time does not require a change in function, making it compatible with the neutral theory of molecular evolution.

In summary, we examined the role of higher-order constraints due to pairwise amino acid interactions on protein sequence evolution properties and found that such interactions result in patterns of amino acid substitution not captured by lower order, independent site models. While the impact of such effects on phylogenetic inference with current methods may not substantially change outcomes at relatively modest sequence divergences (Magee et al. 2021), we show here that such a model begins to provide mechanistic insight into the processes underlying protein sequence evolution.

## Methods

### Data Collection

The relevant data for analysis of the ten protein domain families examined in our study was downloaded from the Datadryad.org data repository provided by de la Paz et al. (2020): https://doi.org/10.5061/dryad.2ngf1vhj8. Pairwise coupling and local field matrices, as well as the starting sequence used in simulations, for each protein domain family were extracted from the “Parameters_orig” MATLAB files available in the repository. The parameter matrices were for 21 amino acid states (20 amino acids + 1 “−” gap character); single nucleotide position and codon matrices were not inferred.

### Potts Hamiltonian Model

Using the SEEC framework created by de la Paz et al. (2020), we simulated protein sequence evolution at the amino acid level under a CE model that includes pairwise epistatic constraints *and* individual site-specific residue preferences, IE model without pairwise epistasis but with individual site-specific preferences, and a UE model of neither pairwise epistasis nor site-specific preferences. SEEC uses a Gibbs sampling approach to simulate sequence evolution by iteratively sampling from a sequence space described by a protein domain family's Potts model (see the methods section in de la Paz et al. 2020). Amino acid changes at a single position are sampled by conditioning on amino acids present in the remaining positions of the protein sequence. The conditional distribution of each of the 21 possible characters (20 amino acids + 1 indel character) is based on their relative fitness in the full sequence, derived from the Potts model:

$$P(a|\;J,h)=\frac{1}{Z}\exp\;\left(\overset{N-1}{\underset{i=1}{\sum }}\;\overset{N}{\underset{\;j=i+1}{\sum }}\;J_{ij}(a_{i},a_{j})+\overset{N}{\underset{i=1}{\sum }}\;h_{i}(a_{i})\right)$$

Here, *i* and *j* are sites in the protein sequence, *Z* is a normalizing constant, *J* is the pairwise site coupling matrix (fig. 1*a*), with *J*_*ij*_(*a*_*i*_, *a*_*j*_) representing the coupling value for sites *i* and *j* when each has residues *a*_*i*_ and *a*_*j*_, respectively. *h* is the local field matrix (fig. 1*b*) with *h*_*i*_(*a*_*i*_) indicating the local field at site *i* when the current residue is *a*_*i*_; *N* is the length of the sequence.

In the CE model, the *J* term is set to the protein domain family-specific coupling constraints, and the *h* term is similarly set to the family-specific site-specific constraints (local fields). The IE model is nested in the CE model with the *J* term is set to 0 for all possible pairs of residues *a*_*i*_ and *a*_*j*_, at all pairs of sites *i* and *j*, such that pairwise epistasis does not contribute to sequence and residue change probabilities. The UE model is nested in the IE model, with the *h* term additionally being set to 0 for all possible residues *a*_*i*_ at all sites *i*.

### Simulations

For each protein domain family analyzed, we simulated 500 replicates of protein sequence evolution for each model (CE, IE, and UE). We initialized the CE, IE, and UE models to have the same random number generator seed to ensure that the same positions were tested for substitutions in each generation across the three models in a given simulation replicate. The same starting sequence, native to each respective protein domain family, was used in all simulations. These “native” sequences were annotated in the MATLAB data files provided by de la Paz et al. (2020) per protein domain family. Each simulation was run for 30,000 generations, and the first 5,000 generations were discarded as burn-in to ensure a steady state. The sequence at the steady state was our reference sequence in each replicate.

Substitutions were then tracked at each position separately. A site was considered invariant as long as the residue did not change from the first generation after burn-in (*I_all_*). While a site can substitute away, then back to the residue found in the first generation and be identical-by-state, we did not consider such sites invariant, as they had successfully accepted a substitution throughout the tracked evolutionary history. We also tracked an adjusted count of I-sites (*I_adj_*), which imposed the following additional criterion to be considered “invariant”: the site must have been randomly selected for a possible substitution sampling at least one time. This adjustment accounted for conditions where a site may appear invariant because it was never randomly selected for substitution testing. Thus, it retained the starting residue state by virtue of the simulation scheme as opposed to rejected substitutions under the Potts Hamiltonian model.

## Data Availability

The scripts used to perform the simulations are available on GitHub at https://github.com/rpatel/SEEC-port-python. The pairwise coupling and site-specific local field parameters required by simulations for ten protein domain families were previously inferred by de la Paz et al. (2020) and were made available at https://doi.org/10.5061/dryad.2ngf1vhj8.
